# Impacts of Groundwater Pumping on Subterranean Microbial Communities in a Deep Aquifer Associated with an Accretionary Prism

**DOI:** 10.3390/microorganisms12040679

**Published:** 2024-03-28

**Authors:** Shinsei Iso, Yu Sato, Hiroyuki Kimura

**Affiliations:** 1 Department of Science, Graduate School of Integrated Science and Technology, Shizuoka University, Shizuoka 422-8529, Japan; 2 Research Center for Thermotolerant Microbial Resources, Yamaguchi University, Yamaguchi 753-8515, Japan; yusato@yamaguchi-u.ac.jp; 3 Department of Geosciences, Faculty of Science, Shizuoka University, Shizuoka 422-8529, Japan; 4 Research Institute of Green Science and Technology, Shizuoka University, Shizuoka 422-8529, Japan

**Keywords:** groundwater flow, deep well, archaeal community, sulfate-reducing archaea, fermentative bacteria, guanine-plus-cytosine content, growth temperature estimation

## Abstract

Accretionary prisms are composed mainly of ancient marine sediment scraped from the subducting oceanic plate at convergent plate boundaries. Anoxic groundwater is stored in deep aquifers associated with accretionary prisms and can be collected via deep wells. We investigated how such groundwater pumping affects the microbial community in a deep aquifer. Groundwater samples were collected from a deep well drilled down to 1500 m every six months (five times in total) after completion of deep well construction and the start of groundwater pumping. Next-generation sequencing and clone-library analyses of 16S rRNA genes were used to describe the subterranean microbial communities in the samples. The archaea: the prokaryote ratio in groundwater increased significantly from 1 to 7% (0 and 7 months after initiating groundwater pumping) to 59 to 72% (13, 19, and 26 months after initiating groundwater pumping), and dominant prokaryotes changed from fermentative bacteria to sulfate-reducing archaea. The optimal growth temperature of the sulfate-reducing archaea, estimated based on the guanine-plus-cytosine contents of their 16S rRNA genes, was 48–52 °C, which agreed well with the groundwater temperature at the deep-well outflow. Our results indicated that, in deep aquifers, groundwater pumping enhances groundwater flow, and the supply of sulfate-containing seawater activates the metabolism of thermophilic sulfate-reducing archaea.

## 1. Introduction

The surface of the Earth is covered with more than ten tectonic plates, including both oceanic and continental plates. Ocean ridges are formed at the boundary region between two ocean plates. A trench is another type of boundary region at the convergence of an oceanic plate and a continental plate. An accretionary prism is a thick layer (more than 10 km thick) of sedimentary material piled up at the plate boundary of an oceanic plate and a continental plate. This material, which was originally deposited on a subducting ocean plate, is accreted onto a non-subducting continental plate during the subduction process [1]. Accretionary prisms are found across large regions of the world, including Alaska and Washington in the United States, New Zealand, Chile, Peru, Indonesia, Taiwan, and Japan [2,3,4,5].

An accretionary prism formed during the Cretaceous (140 Ma to 65 Ma) and Paleogene (65 Ma to 23 Ma) periods is found in Southwest Japan; this is the Shimanto Belt, which is traceable for 1800 km in parallel with the Nankai Trough and Ryukyu Trench [1,2]. The Shimanto Belt originated from ancient marine sediment deposited on the Philippine Sea Plate. The sedimentary layer is composed of permeable sandstone layers and less permeable mudstone layers. The mudstone layers contain abundant organic matter [1,6]. On the other hand, the sandstone layers contain a large amount of anoxic groundwater derived from seawater and/or freshwater, and thus these layers are called aquifers. Seawater and freshwater are cultivated through faults and fracture zones formed by earthquakes occurring at the plate boundaries, forming deep aquifers in an accretionary prism [7,8]. These deep aquifers also store a large amount of natural gas, mainly methane (CH_4_) [9,10,11,12,13,14]. Previous studies indicated that the origin of CH_4_ in natural gas reserves in the deep aquifers is biogenic (formed by methanogenic archaea) or thermogenic (formed by the thermal degradation of organic matter in a mudstone layer) based on stable carbon isotope analysis of CH_4_ and dissolved inorganic carbon (DIC), anaerobic enrichment culture experiments, and DNA analysis [9,10].

Deep wells have been constructed on the accretionary prism in southwest Japan. At some of these wells, anaerobic groundwater in the deep aquifers rises to ground level due to natural water pressure. At others, the groundwater is anaerobically drawn up to ground level by a water pump or gas lift equipment. At spa facilities such groundwater is used as hot spring water. In previous studies, groundwater samples were collected from 23 deep wells constructed on the Shimanto Belt [9,10,12,14]. Illumina sequencing analysis targeting prokaryotic 16S rRNA genes in the groundwater samples revealed the presence of a large number of fermentative bacteria belonging to *Clostridiales*, *Rizobiales*, *Rhodocyclales*, *Bacteroidales*, and *Thermotogales*. Hydrogenotrophic methanogens belonging to *Methanobacteriales*, *Methanomassiliicoccales*, and *Methanomicrobiales* were also detected. In some groundwater samples, acetoclastic methanogens belonging to *Methanosarcinales* were detected. In addition, a high potential of CH_4_ production by hydrogenotrophic methanogens associated with the consumption of molecular hydrogen (H_2_) and carbon dioxide (CO_2_) produced by H_2_-producing fermentative bacteria was observed in the enrichments using the anaerobic groundwater samples amended with organic substrates. These findings suggested the presence of a syntrophic consortium model, in which the anaerobic biodegradation of organic matter in the sediment mediated by H_2_-producing fermentative bacteria and hydrogenotrophic methanogens contributes to CH_4_ production in deep aquifers associated with the accretionary prisms [9,10,12,14]. However, there has been no study on the impact of groundwater pumping on microbial communities in the deep aquifers associated with accretionary prisms.

In this study, groundwater samples were collected from a deep aquifer associated with the accretionary prism approximately every six months beginning right after completion of a deep well of 1500 m depth and the initiation of groundwater pumping. The physicochemical data of groundwater, the composition of natural gas, the metabolic potential of microorganisms, and the microbial community structure were monitored in order to investigate how groundwater pumping affects microbial communities in a deep aquifer.

## 2. Materials and Methods

### 2.1. Study Site and Sample Collection

Groundwater and natural gas were sampled from a deep well (Yaizu Minato no. 1 well; 34°52′25″ N, 138°19′24″ E) that was constructed in January 2021 in the Yaizu area, Shizuoka Prefecture, Japan (Figures S1 and S2). The well was drilled down to 1500 m and constructed from tight steel casing pipes including strainers. It is located 157 m from the coastline of Suruga Bay, bordering the Pacific Ocean. Groundwater and natural gas rise to the ground level due to natural water pressure via the deep well. The capacity of the groundwater pump and the amount of natural gas springing up from the deep well were 36 m^3^ h^−1^ and 63 Nm^3^ h^−1^, respectively. In a previous study, stable carbon isotope analysis of CH_4_ in natural gas and DIC of groundwater suggested that CH_4_ derived from the deep aquifer in the Yaizu area is produced through the non-biological thermal decomposition of organic matter [10].

Groundwater and natural gas were collected on 15 January 2021 (0 months after pumping initiation), 4 August 2021 (7 months after pumping initiation), 8 February 2022 (13 months after the start of pumping), 1 August 2022 (19 months after pumping initiation), and 15 March 2023 (26 months after pumping initiation). Groundwater samples were collected in polycarbonate bottles under an anaerobic condition for chemical and microbiological analyses. The natural gas samples were collected in an inverted funnel underwater and then directed into autoclaved serum bottles according to the method described by Kimura et al. [9].

### 2.2. Measurement of Physical and Chemical Parameters

The physical and chemical parameters of the groundwater were measured at the groundwater outflow of the deep well. Temperature was measured with a CT-460WR thermometer (Custom, Tokyo, Japan). Oxidation-reduction potential (ORP) and pH were measured with RM-20P and HM-20P portable meters (DKK-TOA, Tokyo, Japan), respectively. Electric conductivity (EC) was measured with a CM-21P portable meter (DKK-TOA). Concentrations of natural gas were determined on a GC-2014 gas chromatograph (GC) equipped with a thermal conductivity detector (TCD) and a flame ionization detector (Shimadzu, Kyoto, Japan) following the procedures described by Matsushita et al. [10].

The concentrations of anions (Cl^−^, Br^−^, I^−^, F^−^, PO_4_^3−^, NO_3_^−^, SO_4_^2−^, HCO_3_^−^, acetate, and formate) and cations (Na^+^, Ca^2+^, Mg^2+^, K^+^, and NH_4_^+^) in the groundwater were analyzed with an ICS-1500 ion chromatography system (Dionex, Sunnyvale, CA, USA). Sulfide was analyzed by a sulfide ion detector (Gastech, Ayase-Shi, Kanagawa, Japan). Dissolved organic carbon (DOC) in the groundwater filtered through pre-combusted GF/F glass microfiber filters (GE Healthcare, Buckinghamshire, UK) was measured with a TOC-V total organic carbon analyzer (Shimadzu).

### 2.3. Analysis of Stable Hydrogen and Oxygen Isotopic Ratios

Approximately 30 mL of groundwater sample was collected in polycarbonate bottles for stable isotopic analysis. The stable hydrogen and oxygen isotope ratios of groundwater samples (D/H and ^18^O/^16^O) were measured with a DLT-100 liquid water isotope analyzer (Los Gatos Research, Mountain View, CA, USA) following the procedures described by Dawson et al. [15]. The isotope ratios are reported relative to international standards: Vienna Standard Mean Ocean Water (VSMOW) for δD and δ^18^O. The standard deviations of δD and δ^18^O in groundwater were ±0.5‰ and ±0.1‰, respectively.

### 2.4. Total Cell Count by the Total Direct Count Method

Exactly 10 mL of groundwater sample was filtered using a pre-blackened polycarbonate filter (pore size, 0.22 μm; diameter, 25 mm) (Millipore, Burlington, MA, USA). Microbial cells collected on the filter were stained with SYBR Green I (diluted 100 times) (Life Technologies, Carlsbad, CA, USA) [16,17,18]. Microbial cells were observed under a model BX51 epifluorescence microscope equipped with a U-MNIB3 fluorescence filter (Olympus, Tokyo, Japan), and more than 20 microscopic fields were counted for each sample.

### 2.5. DNA Extraction

To analyze 16S rRNA genes of prokaryotes in groundwater samples, 10 L of groundwater sample was filtered with a Sterivex filter unit (Millipore) using a peristaltic pump Minipuls 3 (Gilson, Middleton, WI, USA). The filter unit was stored at −20 °C. 

DNA extraction from the filter unit was performed using the methods described by Kimura et al. [19]. In brief, microbial cells in the filter unit were lysed using lysozyme and proteinase K solutions. Total nucleotide acid was extracted with successive phenol-chloroform–isoamyl alcohol and chloroform–isoamyl alcohol steps and precipitated with ethanol.

### 2.6. Next-Generation Sequencer (NGS) Analysis of 16S rRNA Genes

The V3–V4 region of archaeal and bacterial 16S rRNA genes was amplified from extracted DNA by PCR using the primer set Pro341F/Pro805R (Table S1). We sequenced 16S rRNA gene amplicons using NGS (Illumina, San Diego, CA, USA). Library generation and sequencing using an Illumina Miseq sequencer was performed according to the method described by Takahashi et al. [20]. Analysis of sequence reads was performed using the Ribosomal Database Project (RDP) Classifier version 2.10 with a confidence threshold of 80% [21]. Sequenced reads were divided into operational taxonomic units (OTUs) sharing more than 97% sequence similarity. In addition, the number of OTUs, coverage, Chao 1, ACE, Shannon index, and Simpson index were calculated using the Quantitative Insights Into Microbial Ecology (QIIME) version 1.5.0 pipeline [22].

### 2.7. Clone Library Analysis of Archaeal 16S rRNA Genes

Archaeal 16S rRNA genes were amplified by PCR from the extracted DNA using an Archaea-specific primer set, 109aF/915aR [23] (Table S1). PCR products of the archaeal 16S rRNA genes were cloned with a Zero Blunt TOPO PCR cloning kit for sequencing with One Shot TOP10 *Escherichia coli* (Life Technologies). The sequences of inserted PCR products selected from recombinant colonies were determined with an Applied Biosystems 3730xl DNA analyzer (Life Technologies).

Chimeric sequences were identified using the Bellerophon chimera detection program [24], and they were excluded from further DNA analysis. Sequences with greater than 97% similarity were determined as distinct OTUs using Genetyx-mac version 22.0.1 (Genetyx, Tokyo, Japan). The coverage of each clone library was calculated using the formula (1 − *n*/*N*), where *n* is the number of OTUs including only one clone, and *N* is the total number of clones [25]. The nearest relative of each OTU was identified by a BLAST search of the National Center for Biotechnology Information (https://blast.ncbi.nlm.nih.gov/Blast.cgi, accessed on 31 October 2023).

### 2.8. Estimation of Growth Temperatures

Previous studies reported that 16S rRNA sequences contain information regarding the thermal features of prokaryotes [19,23,26,27]. These results were based on a high correlation between the guanine-plus-cytosine contents (*P_G+C_*) of the 16S rRNA genes and the growth temperatures of archaea; the *P_G+C_* of the 16S rRNA genes in thermophilic and hyperthermophilic archaea tend to be high, whereas the *P_G+C_* of the 16S rRNA genes in psychrophilic and mesophilic archaea tend to be comparatively low [19]. On the basis of the correlation between the *P_G+C_* of the 16S rRNA genes and the growth temperatures of archaea, Sato et al. [28] proposed linear regression equations to infer minimum (*T_min_*), optimal (*T_opt_*), and maximum growth temperatures (*T_max_*) of cultured and not-yet-cultured archaea.

In this study, the *T_min_*, *T_opt_*, and *T_max_* of archaea detected from the groundwater were estimated using the following equations based on the data from Sato et al. [28].

*T_min_* = 3.91(±0.12)·*P_G+C_* − 201.1(±7.34)

*T_opt_* = 4.24(±0.13)·*P_G+C_* − 202.5(±7.62)

*T_max_* = 4.28(±0.12)·*P_G+C_* − 195.3(±7.11)

In addition, weighted averages of the *P_G+C_* of the 16S rRNA genes were calculated by multiplying the *P_G+C_* of 16S rRNA genes by the number of clones in each OTU and dividing the sum of these values by the total number of clones. Then, the *T_min_*, *T_opt_*, and *T_max_* of the archaeal community for each sample were determined based on the weighted average of the *P_G+C_* of the 16S rRNA genes.

### 2.9. Measurement of Fermentation and CH_4_ Production Potential

Exactly 30 mL of groundwater sample was anoxically injected into an autoclaved 70 mL serum bottle that was then tightly sealed with a sterile butyl rubber stopper and aluminum crimp. To assess the potential for fermentation of microorganisms, groundwater was amended with 3 mL of yeast extract, peptone, and glucose (YPG) medium (10 g yeast extract, 10 g peptone, and 2 g glucose L^−1^ distilled water). The headspaces of serum bottles were filled with pure N_2_ at 150 kPa. The geothermal gradient around the deep well ranges from 25 °C to 30 °C km^−1^ [29]. The mean air temperature in the Yaizu area, Shizuoka Prefecture, Japan, is approximately 18 °C (Japan Meteorological Agency, 2024). Given that the depth of the deep well is 1500 m, the temperature of the deep aquifer is estimated to be 55 °C to 63 °C. Hence, the enrichments were incubated at 55 °C and 65 °C.

To assess the potential for methanogens in groundwater, the groundwater was amended with acetate (20 mM), methanol (20 mM), formate (20 mM), or H_2_/CO_2_ (80:20 [*v*/*v*]; 150 kPa). Except for H_2_/CO_2_-amended bottles, the headspaces of serum bottles were filled with pure N_2_ at 150 kPa. These cultures were anoxically incubated without shaking at 55 °C and 65 °C as described above.

H_2_, CO_2_, and CH_4_ concentrations in the headspaces of serum bottles were measured on a GC-2014 GC equipped with a TCD (Shimadzu) as described above.

## 3. Results

### 3.1. Physicochemical Signatures of Groundwater and Natural Gas

The temperature and pH of the groundwater ranged from 47.0 °C to 51.0 °C and 8.5 to 8.7 (Table 1). ORP and EC ranged from −460 to −252 mV and 2680 to 2950 mS m^−1^, respectively. Componential analysis of the natural gas showed that CH_4_ was present in the natural gas at concentrations ranging from 97.8% to 98.6% (Table 1). 

The concentration ranges were measured for each chemical component, including Na^+^ (3200 to 3510 mg L^−1^), Ca^2+^ (2500 to 2700 mg L^−1^), Mg^2+^ (up to 0.5 mg L^−1^), K^+^ (23 to 25 mg L^−1^), NH_4_^+^ (5.4 to 6.9 mg L^−1^), Cl^−^ (9400 to 10,000 mg L^−1^), Br^−^ (33 to 38 mg L^−1^), I^−^ (6.6 to 6.8 mg L^−1^), HCO_3_^−^ (12 to 30 mg L^−1^), and DOC (up to 1.8 mg L^−1^) (Table S2). F^−^, PO_4_^3−^, NO_3_^−^, SO_4_^2−^, S^2−^, acetate, and formate were found in trace amounts or below the detection limits (Table S2).

### 3.2. Stable Isotopic Signature of Groundwater

Measurements of δD and δ^18^O of groundwater samples were performed. The δD and δ^18^O values of the groundwater ranged from −14.3‰ to −11.7‰ and 0.03‰ to 0.27‰, respectively (Table S3). In order to identify the origin of the groundwater, these values were plotted with VSMOW, local seawater collected at the Yaizu Port situated 157 m from the deep well, and local river water collected from the Setogawa River situated 500 m from the deep well (Figure S3A). The global meteoric water line reported by Craig [30] was also plotted on the δ^18^O versus δD diagram. In the diagram, the δD and δ^18^O values of groundwater were plotted to the right of the global meteoric water line. In addition, the δD values of the groundwater increased gradually from −14.3‰ (0 months after pumping initiation) to −11.8‰ (26 months after the initiation of groundwater pumping) over time after pumping initiation (Figure S3B).

### 3.3. Abundance of Microbial Cells and Diversity of Prokaryotic Communities by NGS Analysis

**Figure 1:**
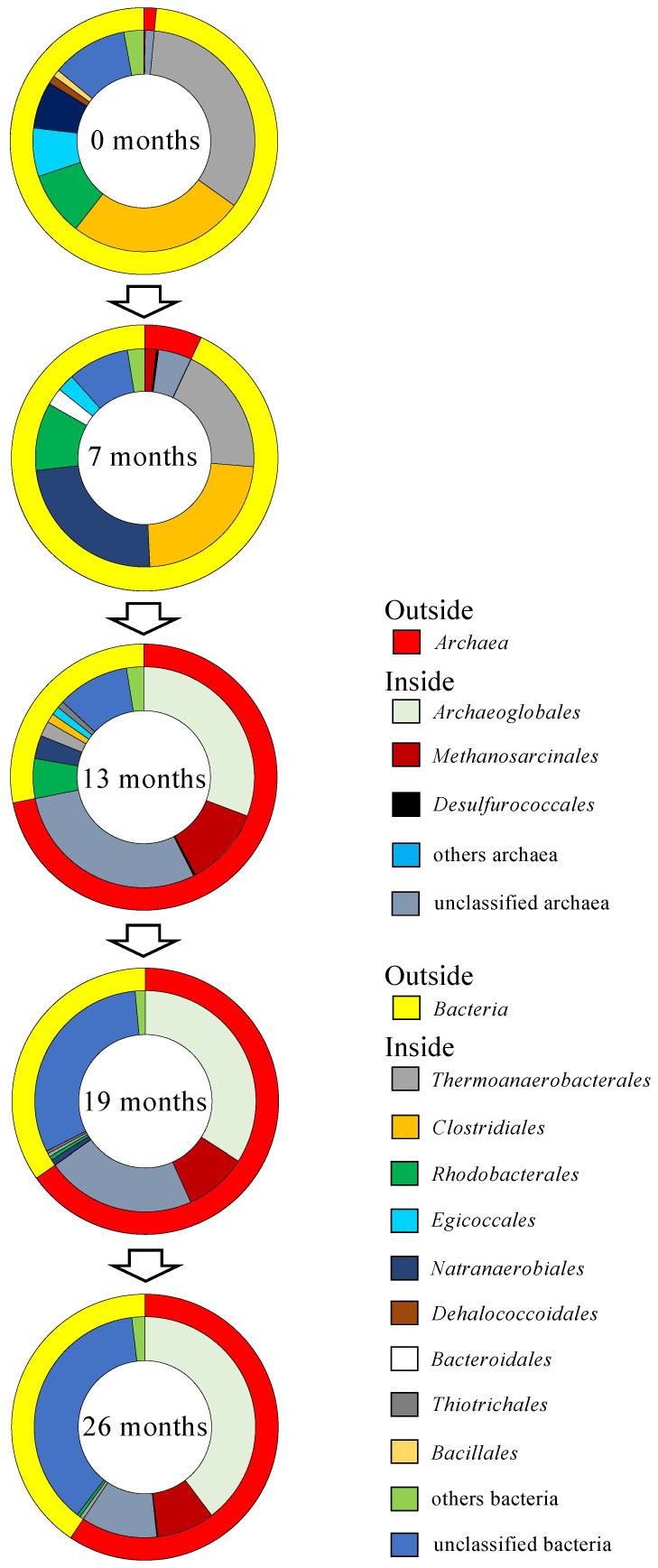
Archaeal and bacterial assemblages in the groundwater. The outside of the doughnut-shaped graph represents the relative abundance of domain *Archaea* and *Bacteria*. The inside represents the relative abundance of orders.

Microbial cell densities in the groundwater collected from the deep well ranged from 2.8 × 10^3^ to 5.6 × 10^4^ cells mL^−1^ (Table 1). Based on NGS analysis of prokaryotic 16S rRNA genes, 11,653 to 32,232 reads and 152 to 465 OTUs were obtained (Table S4). Coverage reached >98.5% in all groundwater samples. The Chao 1 and ACE were 176 to 858 and 185 to 848, respectively. The Shannon and Simpson indexes were 4.29 to 5.32 and 0.908 to 0.944, respectively. Archaeal 16S rRNA genes accounted for only 1.5% and 7.0% of the total reads in the groundwater sampled 0 and 7 months after the start of groundwater pumping, respectively (Figure 1). On the other hand, groundwater sampled at 13, 19, and 26 months after pumping initiation showed that archaeal 16S rRNA genes accounted for 72%, 65%, and 59% of the total reads, respectively. Bacterial 16S rRNA genes accounted for 93% and 98% of the total reads obtained from groundwater sampled 0 and 7 months after the start of groundwater pumping. Meanwhile, 28%, 35%, and 41% of bacterial 16S rRNA genes were obtained from groundwater samples at 13, 19, and 26 months after pumping initiation.

The predominant archaea obtained from groundwater sampled 0 and 7 months after the start of groundwater pumping belonged to unclassified archaea. *Methanosarcinales* and *Desulfurococcales* were also detected as minor members of archaea (Figure 1). By contrast, the most abundant archaea in groundwater sampled 13, 19, and 26 months after pumping initiation belonged to the order *Archaeoglobales*, which can use sulfate or thiosulfate as electron acceptors but cannot grow by fermentation without an electron acceptor [31]. Unclassified archaea and the order *Methanosarcinales* were also detected as minor members of archaea.

The analysis of bacterial 16S rRNA genes revealed that the most dominant bacteria in prokaryotes belong to *Thermoanaerobacterales* and *Clostridiales*, which are known to degrade organic matter to H_2_ and CO_2_ in anoxic environments by fermentation in groundwater sampled 0, 7, and 13 months after pumping initiation (Figure 1) [32,33]. *Natranaerobiales* also dominated in groundwater sampled 7 months after pumping initiation. Some species belonging to the order *Natranaerobiales* are able to use organic matter as an electron donor and thiosulfate as an electron acceptor [34,35]. In addition, *Rhodobacterales*, *Bacillales*, and *Bacteroidales*, which contain some species that can grow by fermentation in anoxic environments, were detected from groundwater sampled 0, 7, and 13 months after the initiation of groundwater pumping [36,37,38]. On the other hand, unclassified bacteria dominated in groundwater sampled 19 and 26 months after pumping initiation.

### 3.4. Archaeal 16S rRNA Genes by Clone Library Analysis and Growth Temperature Estimation

A total of 73 to 92 clones in archaeal 16S rRNA gene clone libraries were sequenced and divided into four to seven OTUs (Table S5). The coverages of the clone libraries ranged between 90.4% and 95.6%. Phylogenetic analysis targeting archaeal 16S rRNA genes revealed the presence of methanogens in all samples, and they were classified into the order *Methanosarcinales* (MNT1_A02), which is able to use H_2_/CO_2_, acetate, mono-, di-, and trimethylamine, and methanol for methanogenesis [39]. The other methanogens were also classified into the orders *Methanobacteriales* and *Methanococcales*, which obtain energy for growth by the oxidation of H_2_ and the reduction of CO_2_ to CH_4_ (MNT1_A05, MNT1_A06, MNT1_A08, and MNT1_A10) [40,41]. In particular, *Methanothrix* belonging to the order *Methanosarcinales*, which uses only acetate for methanogenesis, was dominant in the groundwater sampled 0 and 7 months after pumping initiation (MNT1_A02) [42,43,44]. Phylogenetic analysis of groundwater sampled at 13, 19, and 26 months after pumping initiation also revealed that 66% to 81% of the clones in the library were closely related to the 16S rRNA gene from the cultured organism *Archaeoglobus* (MNT1_A01 and MNT1_A04) (91%, 16S rRNA sequence identity), which uses H_2_ or organic compounds as an electron donor and sulfate or thiosulfate as an electron acceptor during chemoautotrophic or organotrophic growth but cannot grow by fermentation [45]. The results obtained by the clone library analysis were mostly consistent with those obtained from the NGS analysis.

The sequence regions (ca. 800 bp) between the Archaea-specific primer set, 109aF/915aR, were selected, and the approximate growth temperatures were estimated based on the *P_G+C_* of the partial 16S rRNA gene sequences (Table 2). The *P_G+C_* of the internal sequences ranged between 54.7% and 61.2%. The estimated *T_min_*, *T_opt_*, and *T_max_* varied between 12.8(±13.9) °C and 38.2(±14.7) °C, between 29.4(±14.6) °C and 57.0(±15.4) °C, and between 38.8(±13.7) °C and 66.6(±14.5) °C, respectively.

**Figure 2:**
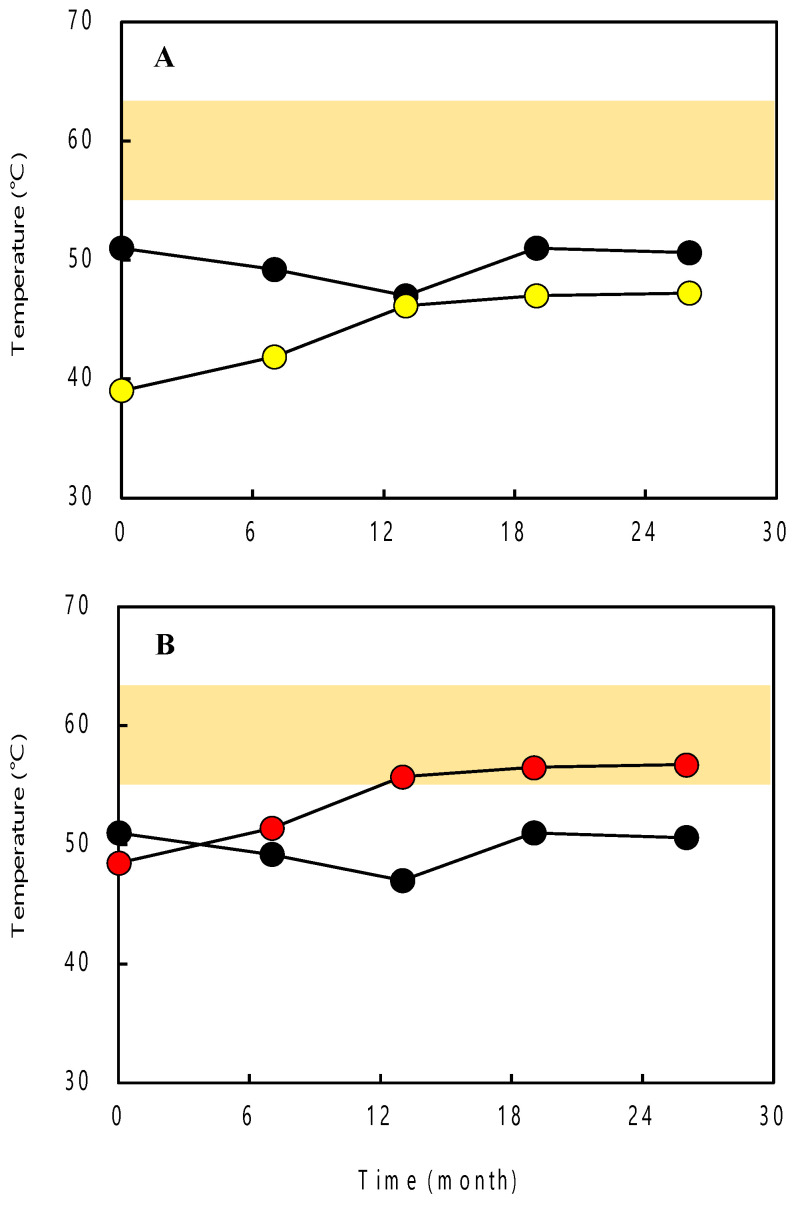
Dynamic of groundwater temperature (black circles) measured at the outflow from the deep well and (**A**) estimated *T_opt_* (yellow circles) and (**B**) estimated *T_max_* (red circles) of the archaeal community for each sample. The colored areas indicate the temperature range of the deep aquifer calculated based on the geothermal gradient and mean air temperature in this study area.

Additionally, the *T_min_*, *T_opt_*, and *T_max_* of the archaeal community for each sample were estimated based on weighted averages of the *P_G+C_* of the 16S rRNA genes. The results showed that these growth temperatures gradually increased with the time elapsed from the start of pumping. Specifically, the estimated *T_min_*, *T_opt_*, and *T_max_* of the archaeal community increased from 21.6(±14.2) °C, 39.0(±14.9) °C, and 48.5(±13.9) °C (0 months after pumping initiation) to 29.7(±14.4) °C, 47.8(±15.1) °C, and 57.4(±14.2) °C (26 months after pumping initiation), respectively (Figure 2).

### 3.5. Potential for H_2_-Producing Fermentative Bacteria and Methanogens

The potential of H_2_-producing fermentative bacteria in the deep aquifer was evaluated through enrichments using groundwater samples amended with YPG medium. As a result, H_2_ and CO_2_ productions by H_2_-producing fermentative bacteria were observed in the enrichments using groundwater sampled 0, 7, and 13 months after the start of groundwater pumping (Figure S4). On the other hand, the H_2_ and CO_2_ productions by fermentation were not confirmed in the enrichments using groundwater sampled 19 and 26 months after pumping initiation. 

The potential of methanogens in the deep aquifer was appraised based on the enrichments using groundwater amended with methanogenic substrates. In the enrichments using H_2_/CO_2_-amended groundwater samples, CH_4_ production by hydrogenotrophic methanogens was not observed. Similarly, CH_4_ production by acetoclastic methanogens and methylotrophic methanogens was not observed in the enrichments using groundwater samples amended with acetate, methanol, formate, or trimethylamine.

## 4. Discussion

The correlation between the physicochemical parameters of groundwater (temperature, pH, ORP, and EC) and time elapsed since the start of groundwater pumping were investigated. The results showed that the physicochemical parameters were not correlated with the time elapsed since pumping initiation (Spearman, *p* > 0.1). These results suggest that groundwater pumping has a limited effect on environmental data in groundwater. The EC values of groundwater samples ranged from 2680 to 2950 mS m^−1^. These values were equivalent to approximately 45% to 50% of the corresponding values in open ocean seawater, which suggests that the deep aquifer located in this study area holds groundwater derived from a mixture of freshwater and seawater that flowed down from the surfaces of the land and seafloor, respectively. The δ^18^O and δD of the groundwater samples ranged from 0.03‰ to 0.27‰ and −14.3‰ to −11.7‰, respectively, and they were plotted to the right of the global meteoric water line in the δ^18^O versus δD diagram (Figure S3A). Previous research has suggested that the main factor contributing to the increase in δ^18^O is the water–rock interaction, e.g., isotopic exchange with ^18^O-rich sedimentary minerals, particularly carbonates [46]. Therefore, the groundwater collected from the deep well may have been affected by water–rock interactions in the thermal deep aquifer associated with the accretionary prism. In addition, the δD values of groundwater gradually increased with time elapsed since the start of groundwater pumping and were approaching that of local seawater rather than local river water (Figure S3B). Furthermore, the Cl^−^ concentration of groundwater tended to gradually increase with time (Table S2). Although a significant change in the EC of the groundwater was not observed, the findings suggest that seawater may affect the deep aquifer in correlation with the time elapsed since pumping initiation.

Based on the NGS analysis, the Chao 1 and ACE of prokaryotes in the deep aquifer were calculated in order to reveal the change in the number of OTUs in the deep aquifer associated with the start of groundwater pumping. The result showed that Chao 1 and ACE were significantly correlated with the time elapsed since pumping initiation (*p* < 0.05). These results may have been due to an increase in the proportions of unclassified archaea and unclassified bacteria, which were present at high levels in the groundwater sampled 13, 19, and 26 months after pumping initiation.

In the groundwater sampled 0 and 7 months after the start of groundwater pumping, the rates of archaea were 1.5% and 7% in prokaryotes (Figure 1). On the other hand, fermentative bacteria belonging to *Thermoanaerobacterales* and *Clostridiales* dominated in prokaryotes (42% and 59%). In addition, *Rhodobacterales*, *Bacillales*, and *Bacteroidales* belonging to the domain *Bacteria* were detected in the groundwater. These members of the bacterial groups possess the ability to grow by fermentation under anoxic environments [36,37,38]. The geothermal gradient around the deep well ranges from 25 °C to 30 °C km^−1^ [29]. The mean air temperature in this study area is approximately 18 °C (Japan Meteorological Agency, 2024). Given that the depth of the deep well is 1500 m, the temperature of the deep aquifer can be calculated as 55 °C to 63 °C. The *T_opt_* of *Thermoanaerobacterales* and *Clostridiales* detected by NGS analysis ranged between 50 °C and 70 °C [47,48,49,50]. It is possible that the temperature condition of the deep aquifer supported the growth of these fermentative bacteria. In addition, the potential of H_2_/CO_2_-producing fermentative bacteria was observed in the enrichments using YPG medium-amended groundwater sampled 0 and 7 months after the start of pumping. Therefore, it is considered that the deep aquifer at 0 and 7 months after groundwater pumping initiation was the ideal environment for the growth of fermentative bacteria because accretionary prisms formed by marine sediments stored large amounts of organic matter derived from phytoplankton and marine organisms that inhabited the ocean in the past [2,51].

Based on NGS analysis, the rates of archaea in groundwater samples at 13 to 26 months after pumping initiation were 59% to 72%, and sulfate-reducing archaea belonging to the order *Archaeoglobales* dominated in prokaryotes (Figure 1). The clone library analysis showed that the clones (MNT1_A01 and MNT1_A04), which were closely related to the 16S rRNA genes of *Archaeoglobus*, dominated at 13, 19, and 26 months after the start of pumping. As mentioned above, the groundwater pumping via the deep well enhances groundwater flow in the deep aquifer, which, in turn, accelerates the permeation of seawater into the deep aquifer. It is therefore considered that the growth of the sulfate-reducing archaea belonging to *Archaeoglobus* was activated by the supply of sulfate derived from seawater. Furthermore, the ability of *Archaeoglobus* to utilize both organic matter and H_2_ as electron donors may also be the reason why sulfate-reducing archaea, rather than sulfate-reducing bacteria, dominated in the thermal deep aquifer. Although sulfate and sulfide related to the sulfate-reducing reaction were below the detection limit in the groundwater samples, in future studies it may be useful to investigate the potential of the sulfate-reducing archaea and the reaction between sedimentary rocks and sulfide.

In clone library analysis, we determined the sequence and *P_G+C_* of archaeal 16S rRNA genes in order to estimate the growth temperatures of archaea. The estimated *T_min_*, *T_opt_*, and *T_max_* of the dominant clones (MNT1_A01 and MNT1_A04) belonging to *Archaeoglobus* at 13, 19, and 26 months after the initiation of groundwater pumping were compared with the temperature of the deep aquifer calculated based on the data of geothermal gradient and mean air temperature in this study area. As a result, their *T_max_* values corresponded well with the temperature of the deep aquifer (Figure 2). A previous study suggested that many of the thermophilic archaea in hot environments, e.g., deep-sea hydrothermal vents and terrestrial hot spring fields, live in a temperature environment that matches their *T_max_* [23]. Therefore, the temperature of the deep aquifer is considered to be a condition under which the sulfate-reducing archaea belonging to *Archaeoglobus* can grow sufficiently.

The rates of H_2_-producing fermentative bacteria belonging to *Thermoanaerobacterales* and *Clostridiales* were decreased from 42 to 59% at 0 and 7 months after pumping initiation to less than 4% at 13, 19, and 26 months after pumping initiation. In the enrichments using groundwater samples amended with YPG medium, high H_2_/CO_2_ production by H_2_-producing fermentative bacteria was observed in the groundwater sampled at 0, 7, and 13 months after groundwater pumping started, whereas H_2_/CO_2_ production was not observed in the enrichments using groundwater sampled at 19 and 26 months after groundwater pumping initiation. *Archaeoglobales*, which was dominant in prokaryotes at 13, 19, and 26 months after the start of groundwater pumping, can utilize H_2_ and organic matter as an electron donor and sulfate as an electron acceptor, but it is not able to grow by fermentation in the absence of electron acceptors [31,45,52,53,54,55]. It is considered that sulfate reduction by sulfate-reducing archaea using organic matter as an electron donor led to competition for organic matter between H_2_-producing fermentative bacteria and the sulfate-reducing archaea in the deep aquifer at 13, 19, and 26 months after pumping initiation. Such competition could have inhibited the growth of H_2_-producing fermentative bacteria belonging to *Thermoanaerobacterales* and *Clostridiales* because fermentative bacteria are thermodynamically less favorable than sulfate-reducing archaea.

In the clone library analysis of samples from the deep aquifer at 13, 19, and 26 months after the start of pumping, *Archaeoglobus* was detected more frequently in the archaeal communities than *Methanothrix*, which can utilize only acetate in the CH_4_ production process. *Archaeoglobus* can utilize acetate as an electron donor for sulfate reduction [55], and another competition for acetate occurs between *Methanothrix* and *Archaeoglobus*. The affinity of sulfate-reducing microorganisms for acetate (−47.6 kJ mol^−1^) is higher than that of acetoclastic methanogens (−31.0 kJ mol^−1^) [56,57,58,59]. In addition, the growth temperature of *Methanothrix* estimated based on the *P_G+C_* of their 16S rRNA genes (MNT1_A02) was much lower than the temperature of groundwater at the outflow from the deep well or the temperature of the deep aquifer calculated based on the geothermal gradient and mean air temperature in the Yaizu area. It is likely that groundwater pumping has facilitated the supply of sulfate-containing seawater to the deep aquifer, which, in turn, has led to a change in the dominant group of the archaeal community from the acetoclastic methanogens to the sulfate-reducing archaea.

Liu et al. [60] suggest the existence of *Candidatus* Methanomixophus hydrogenotrophicum, which is closely related to *Archaeoglobi* containing a group of *Archaeoglobales*. *Ca*. Methanomixophus hydrogenotrophicum lacks the genes encoding dissimilatory sulfate reductase (*dsrAB*) but possesses the genes encoding the coenzyme M methyltransferase complex (*mtrABCDEFGH*) and the genes encoding methyl coenzyme M reductase (*mcrABG*) involved in methanogenic archaea. However, MNT1_A01, MNT1_A04, and MNT1_A09, which were detected in this study and closely related to the 16S rRNA genes from *Archaeoglobales*, were classified into a different cluster from that of *Ca*. Methanomixophus hydrogenotrophicum in the phylogenetic tree based on the 16S rRNA gene sequence (Figure S5). There is a high possibility that CH_4_ production by *Archaeoglobales* did not occur in the deep aquifer.

## 5. Conclusions

A new deep well located in a coastal area offered an opportunity to understand the impact of groundwater pumping via deep wells on the microbial community structure in a deep aquifer associated with an accretionary prism. The results of this analysis showed that the dominant prokaryotes in the deep aquifer changed from fermentative bacteria belonging to *Thermoanaerobacterales* and *Clostridiales* at 0 and 7 months after the start of groundwater pumping to thermophilic sulfate-reducing archaea belonging to *Archaeoglobales* at 13, 19, and 26 months after the start of pumping. Groundwater pumping enhanced groundwater flow and the supply of seawater containing sulfate into the deep aquifer. In addition, the *T_opt_* of archaeal communities estimated based on the *P_G+C_* of the 16S rRNA genes in the deep aquifer at 13, 19, and 26 months after pumping initiation were in good agreement with the groundwater temperatures at the outflow from the deep well. Furthermore, their *T_max_* matched with the temperature of the deep aquifer calculated based on the geothermal gradient and mean air temperature in the area. Taken together, our analyses indicated that the growth of the thermophilic sulfate-reducing archaea in the deep aquifer is facilitated by active groundwater flow within the deep aquifer associated with groundwater pumping.

## Figures and Tables

**Table 1:** Physical and chemical parameters of groundwater, microbial cell density, and components of natural gas.

Date	Groundwater						Natural Gas		
	Temp. (°C)	pH	ORP ^1^ (mV)	EC ^2^ (mS m^−1^)	Cell Density (Cells mL^−1^)		N_2_ (Vol. %)	CH_4_ (Vol. %)	C_2_H_6_ (Vol. %)
0 months after pumping initiation	51.0	8.5	−310	2780	3.0 × 10^3^		1.40	98.6	0.030
7 months after pumping initiation	49.2	8.7	−329	2730	2.1 × 10⁴		1.35	98.6	0.030
13 months after pumping initiation	47.0	8.6	−252	2950	2.8 × 10^3^		1.48	98.5	0.040
19 months after pumping initiation	51.0	8.5	−363	2680	5.6 × 10^4^		2.15	97.8	0.030
26 months after pumping initiation	50.6	8.6	−460	2750	6.9 × 10^3^		2.16	97.8	0.033

^1^ ORP: Oxidation-reduction potential. ^2^ EC: Electric conductivity.

**Table 2:** The growth temperatures of archaea estimated based on the *P_G+C_* of the 16S rRNA genes.

OTU ^1^	Phylogenetic Order	No. of Clones	*P_G+C_* (%)	*T_min_* (°C)	*T_opt_* (°C)	*T_max_* (°C)
0 months after pumping initiation						
MNT1_A02	*Methanosarcinales*	48	56.1	18.3(±14.1)	35.4(±14.8)	44.8(±13.8)
MNT1_A03	*Thermofilales*	11	60.2	34.3(±14.6)	52.7(±15.3)	62.4(±14.3)
MNT1_A05	*Methanobacteriales*	4	54.7	12.8(±13.9)	29.4(±14.6)	38.8(±13.7)
MNT1_A06	*Methanobacteriales*	4	57.9	25.3(±14.3)	43.0(±15.0)	52.5(±14.1)
MNT1_A08	*Methanococcales*	3	56.5	19.8(±14.1)	37.1(±14.8)	46.5(±13.9)
MNT1_A09	*Archaeoglobales*	2	61.2	38.2(±14.7)	57.0(±15.4)	66.6(±14.5)
MNT1_A07	*Thermofilales*	1	60.6	35.8(±14.6)	54.4(±15.3)	64.1(±14.4)
			57.0 ^2^	21.6(±14.2) ^3^	39.0(±14.9) ^3^	48.5(±13.9) ^3^
7 months after pumping initiation						
MNT1_A02	*Methanosarcniales*	52	56.1	18.3(±14.1)	35.4(±14.8)	44.8(±13.8)
MNT1_A01	*Archaeoglobales*	24	59.9	33.1(±14.5)	51.5(±15.3)	61.1(±14.3)
MNT1_A03	*Thermofilales*	9	60.2	34.3(±14.6)	52.7(±15.3)	62.4(±14.3)
MNT1_A04	*Archaeoglobales*	3	59.2	30.4(±14.4)	48.5(±15.2)	58.1(±14.2)
MNT1_A10	*Methanococcales*	2	58.1	26.1(±14.3)	43.8(±15.0)	53.4(±14.1)
			57.7 ^2^	24.4(±14.3) ^3^	42.0(±15.0) ^3^	51.5(±14.0) ^3^
13 months after pumping initiation						
MNT1_A01	*Archaeoglobales*	52	59.9	33.1(±14.5)	51.5(±15.3)	61.1(±14.3)
MNT1_A02	*Methanosarcinales*	29	56.1	18.3(±14.1)	35.4(±14.8)	44.8(±13.8)
MNT1_A04	*Archaeoglobales*	9	59.2	30.4(±14.4)	48.5(±15.2)	58.1(±14.2)
MNT1_A03	*Thermofilales*	2	60.2	34.3(±14.6)	52.7(±15.3)	62.4(±14.3)
			58.6 ^2^	28.2(±14.4) ^3^	46.2(±15.1) ^3^	55.7(±14.1) ^3^
19 months after pumping initiation						
MNT1_A01	*Archaeoglobales*	53	59.9	33.1(±14.5)	51.5(±15.3)	61.1(±14.3)
MNT1_A02	*Methanosarcinales*	17	56.1	18.3(±14.1)	35.4(±14.8)	44.8(±13.8)
MNT1_A04	*Archaeoglobales*	14	59.2	30.4(±14.4)	48.5(±15.2)	58.1(±14.2)
MNT1_A03	*Thermofilales*	1	60.2	34.3(±14.6)	52.7(±15.3)	62.4(±14.3)
			59.0 ^2^	29.7(±14.4) ^3^	47.8(±15.1) ^3^	57.4(±14.2) ^3^
26 months after pumping initiation						
MNT1_A01	*Archaeoglobales*	36	59.9	33.1(±14.5)	51.5(±15.3)	61.1(±14.3)
MNT1_A04	*Archaeoglobales*	35	59.2	30.4(±14.4)	48.5(±15.2)	58.1(±14.2)
MNT1_A02	*Methanosarcinales*	16	56.1	18.3(±14.1)	35.4(±14.8)	44.8(±13.8)
MNT1_A07	*Thermofilales*	2	60.6	35.8(±14.6)	54.4(±15.3)	64.1(±14.4)
			59.0 ^2^	29.7(±14.4) ^3^	47.8(±15.1) ^3^	57.4(±14.2) ^3^

^1^ OTU: operational taxonomic unit. ^2^ Weighted average was calculated by multiplying *P_G+C_* of 16S rRNA gene by the number of clones in each OTU and dividing the sum of these values by the total number of clones. ^3^ The growth temperature of the archaeal community for each sample was determined based on the weighted average of the *P_G+C_* of the 16S rRNA genes.

## Data Availability

The 16S rRNA gene sequences obtained from NGS analysis have been deposited in the DDBJ/ENA/GenBank database under accession numbers SAMD00633944, SAMD00633945, and DRA016801. The 16S rRNA gene sequences obtained from clone library analysis were deposited in the DDBJ/ENA/GenBank database under accession numbers LC796997 to LC797006.

## Supplementary Materials

The following supporting information can be downloaded at: https://www.mdpi.com/article/10.3390/microorganisms12040679/s1, Figure S1: Geological map of Southwest Japan. The location of the accretionary prism known as the Shimanto Belt is according to Kano et al. [2]. MTL, Median Tectonic Line; ISTL, Itoigawa-Shizuoka Tectonic Line; Figure S2: Photographs of the deep well “Yaizu Minato no.1 well” located in Shizuoka Prefecture, Japan. The area enclosed in a square indicates the deep well for sample collection; Figure S3: (A) Stable hydrogen and oxygen isotopic compositions of the groundwater samples (open circles) along with those of Vienna Standard Mean Ocean Water (VSMOW) (black square), local seawater (open square), and river water (open diamond). The broken line represents the global meteoric water line [30]. (B) This figure shows the differences in stable hydrogen and oxygen isotope ratios of each sample in detail. The numbers filled in the plots indicate elapsed time (month) since the start of groundwater pumping; Figure S4: Biogas productions from the groundwater (GW) amened with YPG medium. Cumulative measurements in the gas phase of bottled cultures are shown as follows: H_2_ (▲); CH_4_ (○); CO_2_ (×). Incubation temperature is mentioned in the figure; Figure S5: Phylogenetic tree based on 16S rRNA gene sequences derived from the groundwater samples. Accession numbers are shown in parentheses. Bootstrap values determined from 1000 iterations are indicated at branching points; Table S1: Primers for PCR used in this study; Table S2: Chemical characteristic of the groundwater samples and normal seawater (mg L^−1^); Table S3: Stable isotopic signature of the groundwater samples; Table S4: Number of prokaryotic 16S rRNA gene sequences derived from the groundwater samples and statistical estimators; Table S5: Archaeal 16S rRNA gene sequences derived from the groundwater samples. References [61,62,63] are cited in the Supplementary Materials.
