# Supplementary material for: Impacts of Groundwater Pumping on Subterranean Microbial Communities in a Deep Aquifer Associated with an Accretionary Prism
Source: Microorganisms. 2024 Mar 28;12(4):679. doi: 10.3390/microorganisms12040679 (PMC11052133; doi:10.3390/microorganisms12040679)
Supplement: Supplementary file 1 [file microorganisms-12-00679-s001.zip › microorganisms-2921174-new supplementary.pdf]

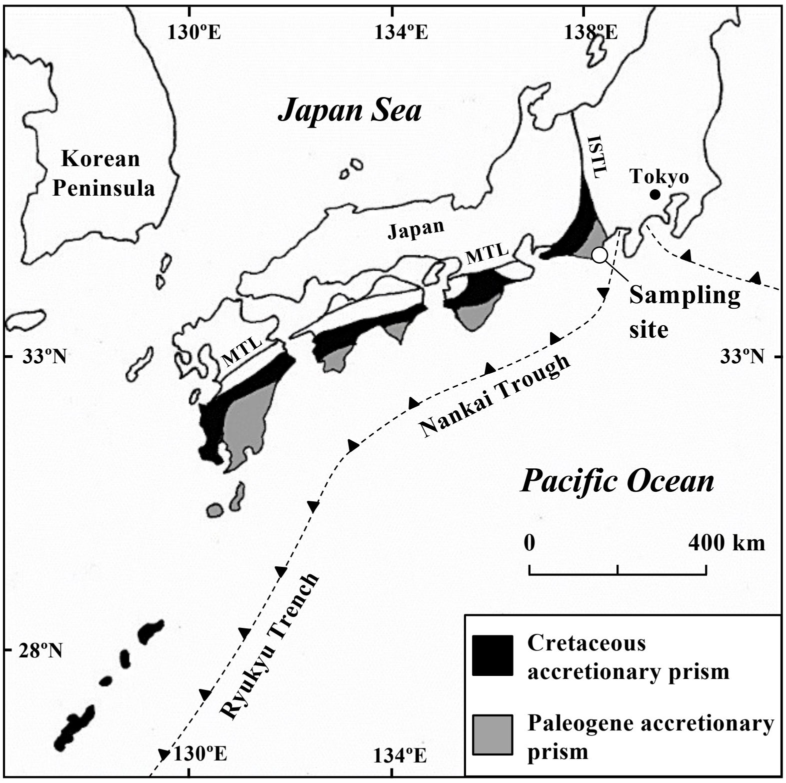

Figure S1. Geological map of Southwest Japan. The location of the accretionary prism known as the Shimanto Belt is according to Kano et al. [2]. MTL, Median Tectonic Line; ISTL, Itoigawa-Shizuoka Tectonic Line.

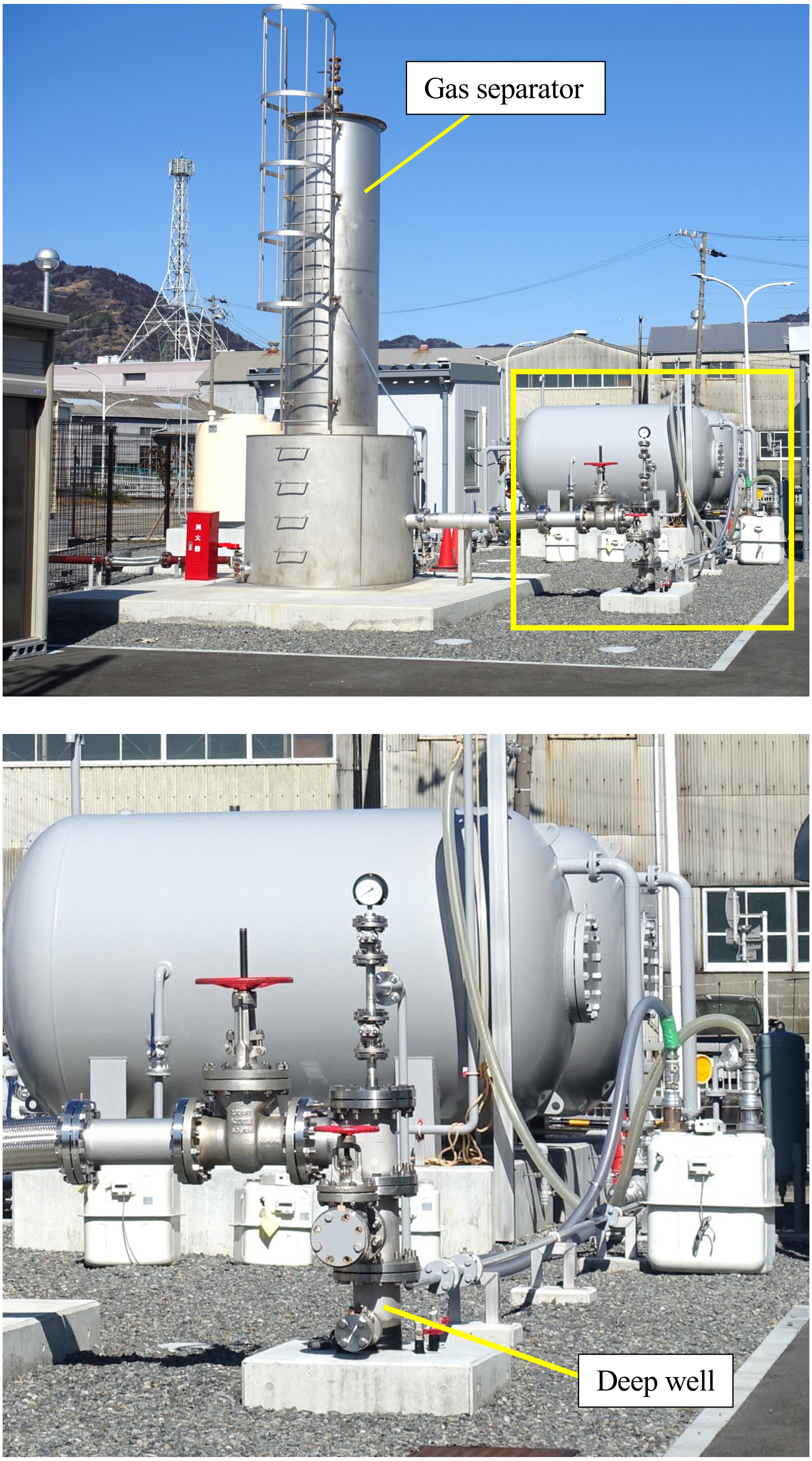

Figure S2. Photographs of the deep well “Yaizu Minato no.1 well” located in Shizuoka Prefecture, Japan. The area enclosed in a square indicates the deep well for sample collection.

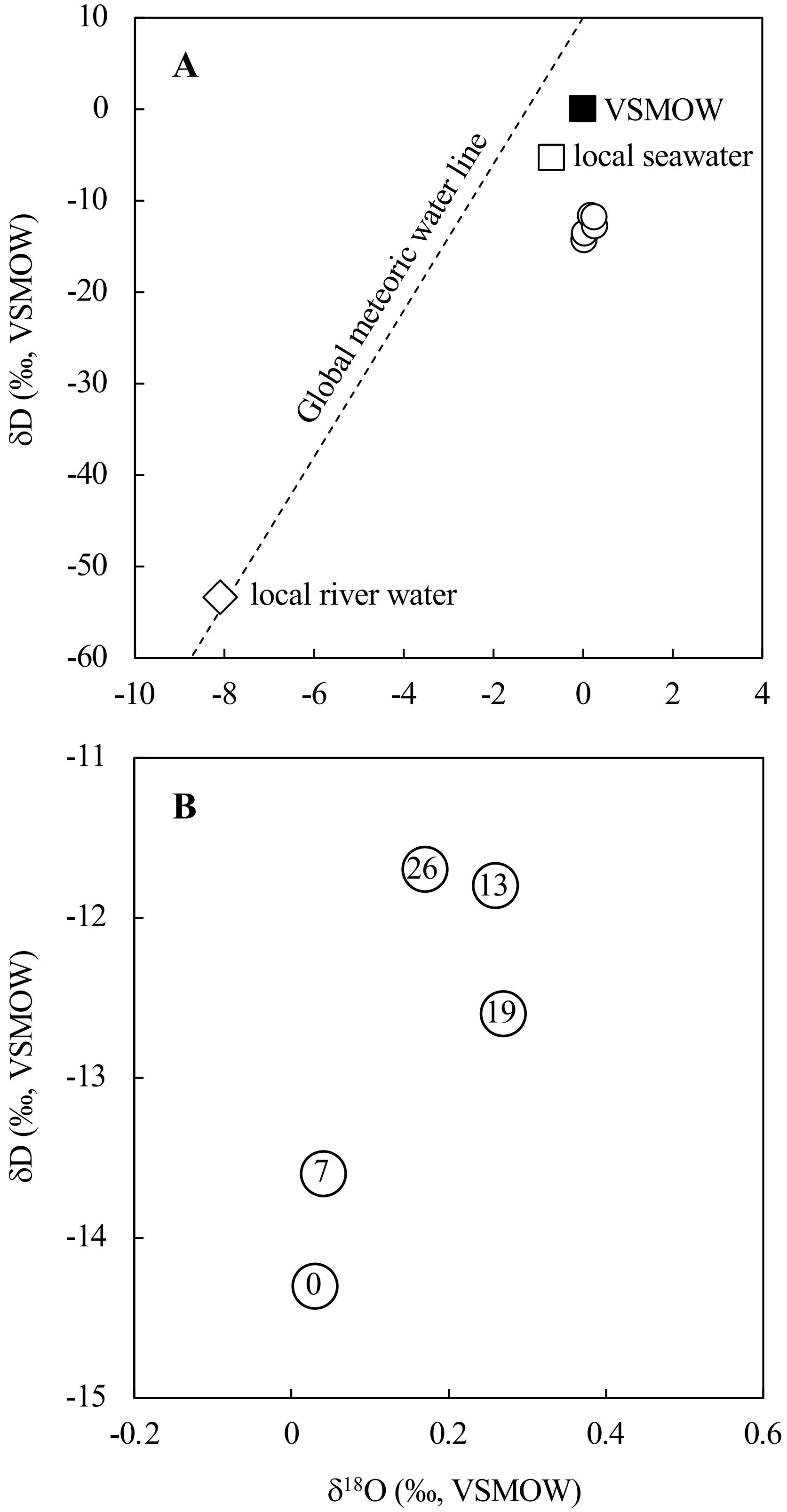

Figure S3. (A) Stable hydrogen and oxygen isotopic compositions of the groundwater samples (open circles) along with those of Vienna Standard Mean Ocean Water (VSMOW) (black square), local seawater (open square), and local river water (open diamond). The broken line represents the global meteoric water line [30]. (B) This figure shows the differences in stable hydrogen and oxygen isotope ratios of each sample in detail. The numbers filled in the plots indicate elapsed time (month) since the start of groundwater pumping.

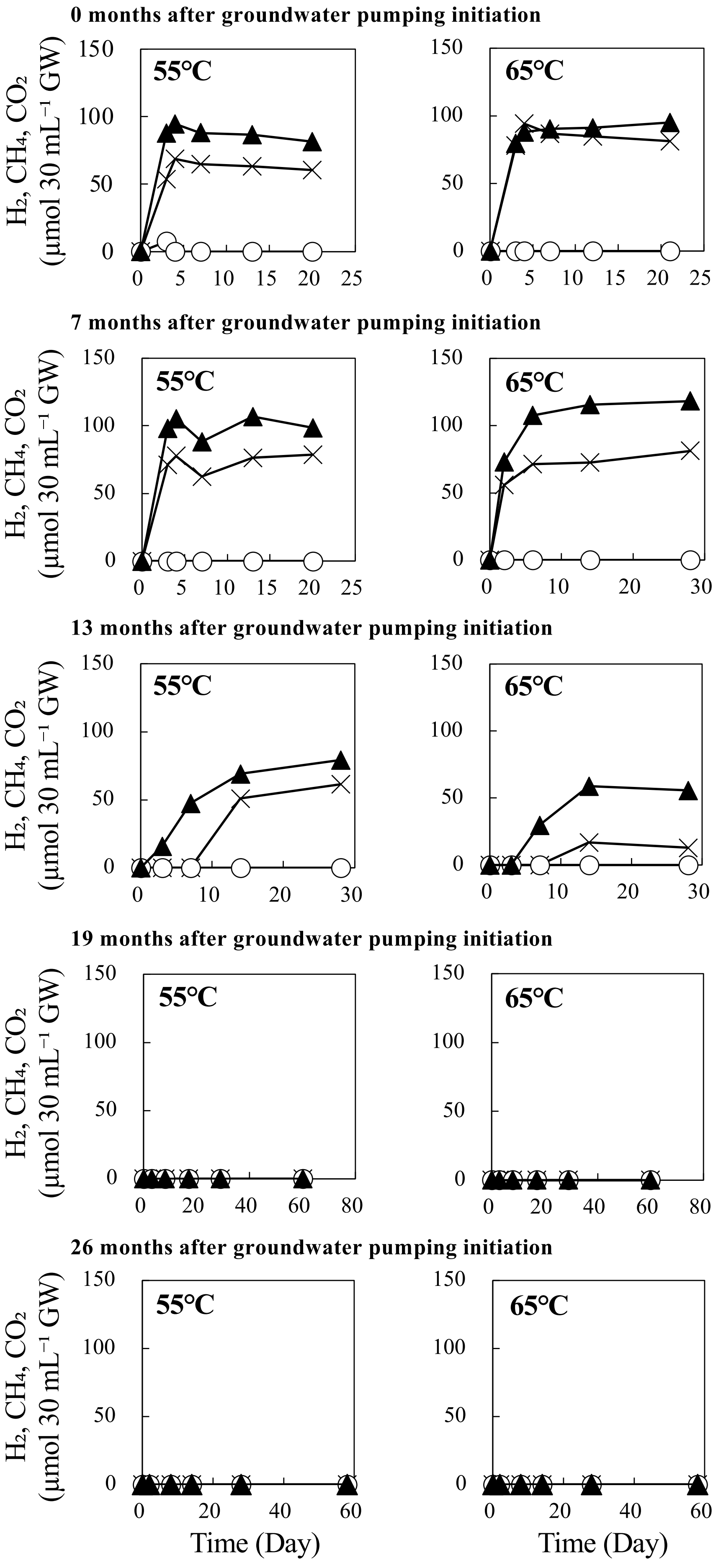

Figure S4. Biogas productions from the groundwater (GW) amened with YPG medium. Cumulative measurements in the gas phase of bottled cultures are shown as follows: H_2 (▲); CH_4 (○); CO_2 (×). Incubation temperature is mentioned in the figure.

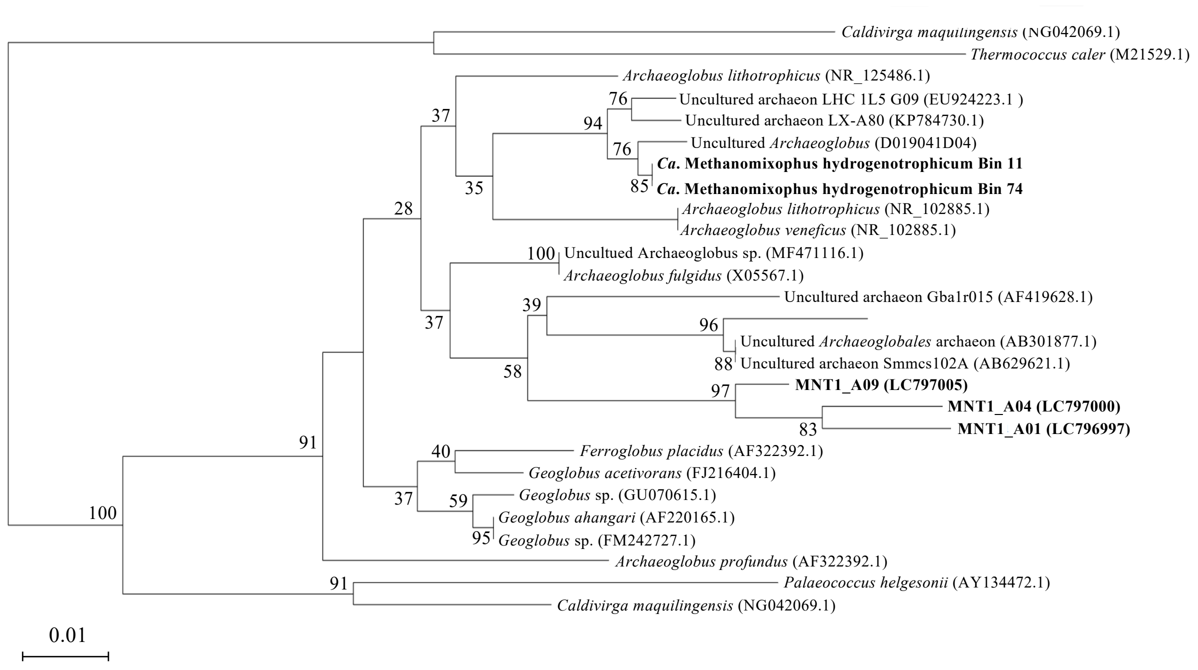

Figure S5. Phylogenetic tree based on 16S rRNA gene sequences derived from the groundwater samples. Accession numbers are shown in parentheses. Bootstrap values determined from 1000 iterations are indicated at branching points.

Table S1. Primers for PCR used in this study.

Primer	Sequences	Target	References
109aF	5’-AMD GCT CAG TAA CAC GT-3’	Archaeal 16S rRNA gene	Groβkopf et al. [61]
915aR	5’-GTG CTC CCC CGC CAA TTC CT-3’	Archaeal 16S rRNA gene	Groβkopf et al. [61]
Pro341F	5’-AAT GAT ACG GCG ACC ACC GAG ATC TAC ACT CTT TCC CTA CAC GAC GCT CTT CCG ATC TCC TAC GGG AGG CAG CAG CCT ACG GGN BGC ASC AG-3’	Prokaryotic 16S rRNA gene	Takahashi et al. [20]
Pro805R	5’ -CAA GCA GAA GAC GGC ATA CGA GAT NNN NNN GTG ACT GGA GTT CAG ACG TGT GCT CTT CCG ATC TGA CTA CNV GGG TAT CTA ATC C- 3’	Prokaryotic 16S rRNA gene	Takahashi et al. [20]

Table S2. Chemical characteristic of the groundwater samples and normal seawater (mg L^{-1}).

Chemical property	0 months after pumping initiation	7 months after pumping initiation	13 months after pumping initiation	19 months after pumping initiation	26 months after pumping initiation	Normal Seawater[3]
Na^+	3,500	3,400	3,200	3,400	3,510	10,781
Ca^{2+}	2,700	2,600	2,500	2,600	2,650	412
Mg^{2+}	0.5	b.d.[2]	0.5	b.d.[2]	0.2	1,283
K^+	23	24	24	23	25	399
NH_4^+	6.0	6.9	6.1	6.9	5.4	n.a.[4]
Cl^-	9,400	9,400	9,900	9,500	10,000	19,352
Br^-	35	33	38	36	35	67
I^-	6.6	6.8	6.8	6.8	n.a.[4]	0.05
F^-	b.d.[2]	b.d.[2]	b.d.[2]	b.d.[2]	0.10	n.a.[4]
PO_4^{3-}	b.d.[2]	b.d.[2]	b.d.[2]	b.d.[2]	b.d.[2]	n.a.[4]
NO_3^-	b.d.[2]	b.d.[2]	b.d.[2]	b.d.[2]	b.d.[2]	n.a.[4]
SO_4^{2-}	b.d.[2]	b.d.[2]	b.d.[2]	b.d.[2]	b.d.[2]	2,712
S^{2-}	b.d.[2]	b.d.[2]	b.d.[2]	b.d.[2]	b.d.[2]	b.d.[2]
HCO_3^-	20	12	15	24	30	105
acetate	b.d.[2]	b.d.[2]	b.d.[2]	b.d.[2]	b.d.[2]	n.a.[4]
formate	b.d.[2]	b.d.[2]	b.d.[2]	b.d.[2]	b.d.[2]	n.a.[4]
DOC[1]	0.9	1.8	0.9	0.4	b.d.[2]	1.4-2.6[5]

[1] DOC: Dissolved organic carbon.

[2] b.d.: Below detection limit.

[3] Normal seawater data is referred to Millero et al. [62]

[4] n.a.: Not analyzed.

[5] DOC value in normal seawater is referred to Kimura et al. [63]

Table S3. Stable isotopic signature of the groundwater samples.

Date	δD (‰, VSMOW[1])	$\delta^{18}O$ (‰, VSMOW[1])
0 months after pumping initiation	-14.3	0.03
7 months after pumping initiation	-13.6	0.04
13 months after pumping initiation	-11.7	0.17
19 months after pumping initiation	-12.8	0.27
26 months after pumping initiation	-11.8	0.26

[1]VSMOW: Vienna Standard Mean Ocean Water.

Table S4. Number of prokaryotic 16S rRNA gene sequences derived from the groundwater samples and statistical estimators.

Date	Total of reads	No. of OTUs[1]	Coverage (%)	Chao 1	ACE	Shannon index	Simpson index
0 months after pumping initiation	13,936	152	99.8	176	185	4.73	0.932
7 months after pumping initiation	32,232	262	99.7	456	431	4.29	0.908
13 months after pumping initiation	21,486	378	99.5	550	532	5.32	0.944
19 months after pumping initiation	20,436	465	99.6	680	660	4.85	0.918
26 months after pumping initiation	11,653	419	98.5	858	848	5.24	0.932

[1] OTUs: operational taxonomic units.

Table S5. Archaeal 16S rRNA gene sequences derived from the groundwater samples.

OTU[1]	No. of clones	Phylogenetic order	Closest cultivated species (%, identity)
0 months after pumping initiation			
MNT1_A02	48	*Methanosarcinales*	*Methanothrix harundinacea* (88)
MNT1_A03	11	*Thermofilales*	*Thermofilum adornatum* (84)
MNT1_A05	4	*Methanobacteriales*	*Methanobacterium alkalithermotolerans* (100)
MNT1_A06	4	*Methanobacteriales*	*Methanothermobacter marburgensis* (99)
MNT1_A08	3	*Methanococcales*	*Methanotorris igneus* (81)
MNT1_A09	2	*Archaeoglobales*	*Archaeoglobus veneficus* (92)
MNT1_A07	1	*Thermofilales*	*Thermofilum adornatum* (85)
Total	73	(Coverage = 90.4%)	
7 months after pumping initiation			
MNT1_A02	52	*Methanosarcinales*	*Methanothrix harundinacea* (88)
MNT1_A01	24	*Archaeoglobales*	*Archaeoglobus veneficus* (91)
MNT1_A03	9	*Thermofilales*	*Thermofilum adornatum* (84)
MNT1_A04	3	*Archaeoglobales*	*Archaeoglobus veneficus* (91)
MNT1_A10	2	*Methanococcales*	*Methanocaldococcus lauensis* (77)
Total	90	(Coverage = 94.0%)	
13 months after pumping initiation			
MNT1_A01	52	*Archaeoglobales*	*Archaeoglobus veneficus* (91)
MNT1_A02	29	*Methanosarcinales*	*Methanothrix harundinacea* (88)
MNT1_A04	9	*Archaeoglobales*	*Archaeoglobus veneficus* (91)
MNT1_A03	2	*Thermofilales*	*Thermofilum adornatum* (84)
Total	92	(Coverage = 95.6%)	
19 months after pumping initiation			
MNT1_A01	53	*Archaeoglobales*	*Archaeoglobus veneficus* (91)
MNT1_A02	17	*Methanosarcinales*	*Methanothrix harundinacea* (88)
MNT1_A04	14	*Archaeoglobales*	*Archaeoglobus veneficus* (91)
MNT1_A03	1	*Thermofilales*	*Thermofilum adornatum* (84)
Total	85	(Coverage = 95.3%)	
26 months after pumping initiation			
MNT1_A01	36	*Archaeoglobales*	*Archaeoglobus veneficus* (91)
MNT1_A04	35	*Archaeoglobales*	*Archaeoglobus veneficus* (91)
MNT1_A02	16	*Methanosarcinales*	*Methanothrix harundinacea* (87)
MNT1_A07	2	*Thermofilales*	*Thermofilum adornatum* (85)
Total	89	(Coverage = 95.5%)	

[1]OTU: operational taxonomic unit.
